# Mutation in histone deacetylase HDA-3 leads to shortened locomotor healthspan in *Caenorhabditis elegans*

**DOI:** 10.18632/aging.202296

**Published:** 2020-12-03

**Authors:** Kazuto Kawamura, Ichiro N. Maruyama

**Affiliations:** 1 Information Processing Biology Unit, Okinawa Institute of Science and Technology Graduate University, Onna-son, Kunigami-gun, Okinawa, Japan

**Keywords:** *hda-3*, age-related locomotor impairment, BATH domain, aging, longevity

## Abstract

Some genes are essential for survival, while other genes play modulatory roles on health and survival. Genes that play modulatory roles may promote an organism’s survival and health by fine-tuning physiological processes. An unbiased search for genes that alter an organism’s ability to maintain aspects of health may uncover modulators of lifespan and healthspan. From an unbiased screen for *Caenorhabditis elegans* mutants that show a progressive decline in motility, we aimed to identify genes that play a modulatory role in maintenance of locomotor healthspan. Here we report the involvement of *hda-3,* encoding a class I histone deacetylase, as a genetic factor that contributes in the maintenance of general health and locomotion in *C. elegans*. We identified a missense mutation in HDA-3 as the causative mutation in one of the isolated strains that show a progressive decline in maximum velocity and travel distance. From transcriptome analysis, we found a cluster of genes on Chromosome II carrying BATH domains that were downregulated by *hda-3*. Furthermore, downregulation of individual *bath* genes leads to significant decline in motility. Our study identifies genetic factors that modulate the maintenance of locomotor healthspan and may reveal potential targets for delaying age-related locomotor decline.

## INTRODUCTION

Mutations of some genes cause severe defects in an organism’s health, while mutations of other genes cause subtle effects on health. Genes with modulatory effects may be involved in how well an organism can maintain its health and how long an organism can survive. One aspect of health that determines quality of life in the elderly is locomotor ability [1]. Age-related decline in locomotor ability is a predictor of loss of independence, depressive symptoms, morbidity, and mortality [2]. A better understanding of the factors that contribute to the maintenance of locomotor ability may enable novel approaches to prevent or delay age-related decline in locomotor ability and general health.

The nematode *Caenorhabditis elegans* provides a unique opportunity to study locomotor healthspan due to the availability of genetic techniques and its short lifespan of approximately two weeks [3]. From a candidate-based screen of genes that extend the swimming ability of aged *C. elegans* worms, the epidermal growth factor signalling pathway was found to extend locomotor healthspan and lifespan [4]. Recently, loss-of-function mutations in the calcium-activated potassium channel, SLO-1, were found to improve locomotor healthspan and lifespan in *C. elegans* [5].

In Sydney Brenner’s seminal forward genetic screen using *C. elegans*, he isolated uncoordinated (*unc*) mutants and identified genes that are required for the proper development of locomotor ability [3]. In order to identify novel genetic factors that play modulatory roles in the maintenance of locomotor healthspan, we previously carried out an unbiased forward genetic screen and isolated five *C. elegans* mutants that show progressive decline in motility [6].

In the present study, we analyzed one of the isolated strains from the screen, OF1262, to identify genes that modulate the maintenance of locomotor healthspan. From analyzing this strain, we found that a missense mutation in HDA-3 leads to reduced health, including progressive decline in maximum velocity, travel distance, and reduced longevity. HDA-3 is a *C. elegans* ortholog of human class I histone deacetylases (HDACs) [7]. *C. elegans* class I HDACs have been found to play roles in both development and aging-related pathologies. Loss of HDA-1 function leads to disorganized gonad structures and aberrant vulval development such as protruded vulva or the development of multiple vulvae [8, 9]. *C. elegans* class I HDACs have also been found to play a role in polyglutamine toxicity and longevity [10, 11]. The missense mutation identified in the OF1262 strain is a glycine to glutamic acid substitution at the 271^st^ amino acid (G271E) in HDA-3. This mutation causes a gene cluster carrying BATH domains to be transcriptionally repressed. Proper regulation of gene expression by HDA-3 may be required for full maintenance of locomotor healthspan and longevity during adulthood.

## RESULTS

### OF1262 strain that shows shortened adult locomotor healthspan carries *hda-3(ix241)* and *dys-1(ix259)* mutations

**Figure 1:**
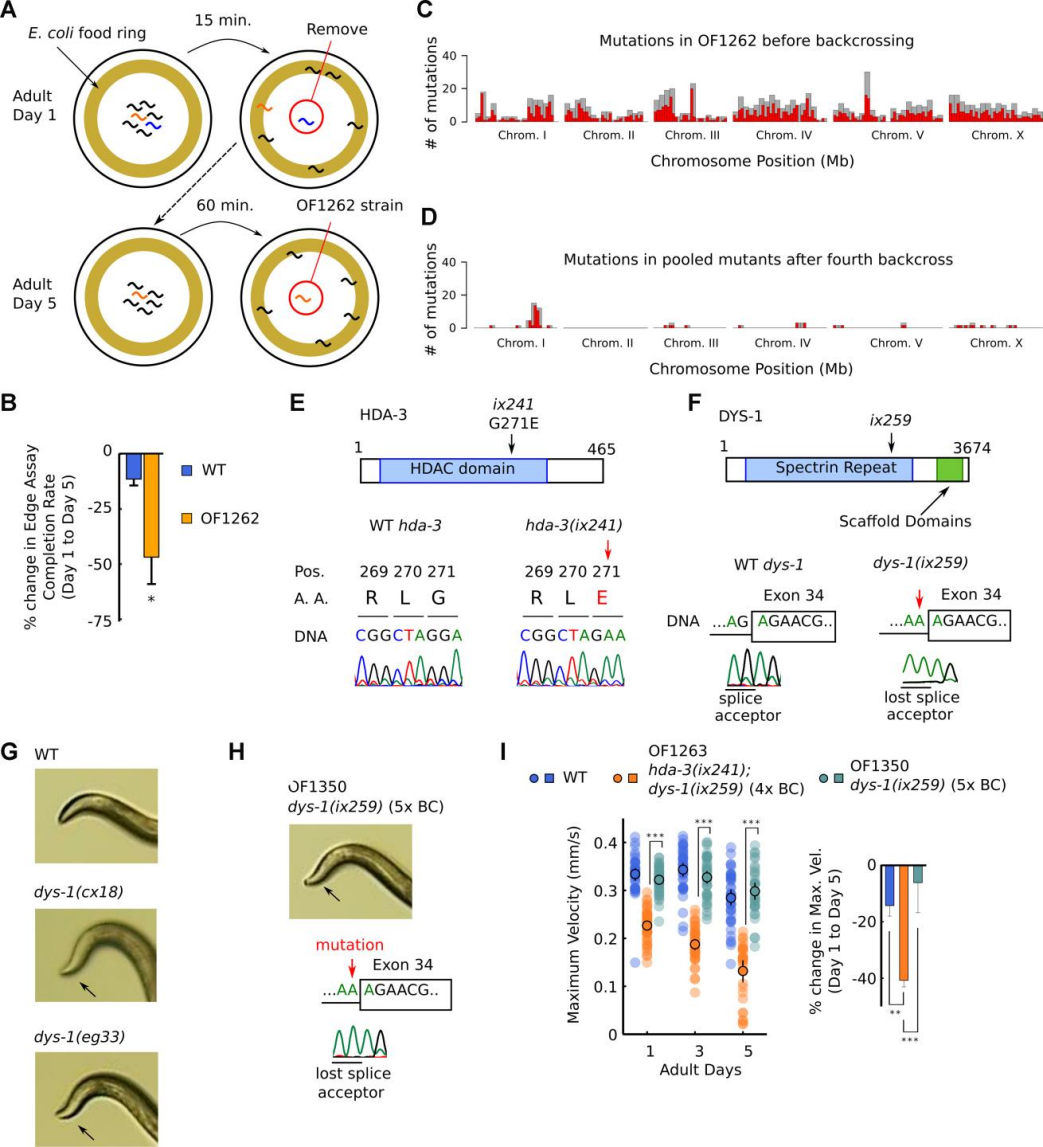
**OF1262 strain shows locomotor deficit and carries *hda-3(ix241)* and *dys-1(ix259)* mutations.** (**A**) Schematic of forward genetic screen to isolate OF1262 strain, a strain that shows progressive decline in adult motility. (**B**) Percent change in completion rate of the Edge Assay on adult day 5 compared to adult day 1. N = 3 biological replicate plates (100 worms or more per plate on adult day 1). (**C**) Mutation frequency along each chromosome for the OF1262 strain. Red bars indicate 0.5-Mb bins and grey bars indicate 1.0-Mb bins. (**D**) Mutation frequency along each chromosome for remaining mutations after subtracting mutations found in backcrossed strains that did not show a progressive decline in locomotor ability from mutations found in backcrossed strains that showed a progressive decline in locomotor ability. (**E**) Effect of *hda-3(ix241)* mutation on DNA sequence and amino acid sequence. (**F**) Effect of *dys-1(ix259)* mutation on DNA sequence. (**G**) Photos of head curvature during forward crawling in WT, *dys-1(cx18)* and *dys-1(eg33)* worms. Exaggerated head bending indicated by arrows. (**H**) (*Top*) Photo of head curvature during forward crawling in OF1350 *dys-1(ix259)* worms. (*Bottom*) DNA sequence of *dys-1*(*ix259*) mutation site. (**I**) (*Left*) Maximum velocities of WT, OF1263 *hda-3(ix241);dys-1(ix259)* (4x BC)*,* and OF1350 *dys-1(ix259)* (5x BC) worms. N = 30–45 worms per strain for each day (10–15 worms from 3 biological replicate plates). (*Right*) Percent change in maximum velocity of WT, OF1263*,* and OF1350 worms on adult day 5 compared to adult day 1. N = 3 biological replicate plates. ***p < 0.001; **p < 0.01.

Previously, we carried out a forward genetic screen for *C. elegans* mutants with a shortened adult locomotor healthspan [6]. One of the isolated strains was the OF1262 strain which showed a slight developmental deficit and a marked progressive decline in the ability to reach a food source located on the outer edge of an agar plate (Figure 1A, 1B) [6]. The OF1262 strain shows a progressive decline in maximum velocity and travel distance during adulthood as measured by 1.0-minute video recordings of worms moving on an agar plate without food. OF1262 worms show a progressive decline in maximum velocity and travel distance even when measured on a plate without a food cue, suggesting the decline is likely not due to defects in the sensory system. Maximum velocity is the highest velocity that the worm reaches during the 1.0-minute video recording, while travel distance is the total distance that the worm covers. Together, these measures estimate the maximum neuromuscular capacity of worms, as well as the capacity to maintain a certain level of locomotion. In all of the strains we tested, we observed similar trends between maximum velocity and travel distance, and thus display only the maximum velocity measurements in the main figures and show the travel distance measurements in the supplementary figures. We observed variations in both measurements among separate experiments, suggesting sensitivity of the assay to slight differences in culture conditions such as temperature and humidity.

In order to identify the causative mutation site that leads to progressive decline in maximum velocity and travel distance in the OF1262 strain, we carried out whole genome sequencing in strains that showed and did not show the progressive decline after backcrossing. We identified mutations that were shared among the genomes of backcrossed strains that showed the progressive decline in maximum velocity, and subtracted the shared mutations among backcrossed strains that did not show the progressive decline in maximum velocity. A peak of mutations remained on Chromosome I (Figure 1C, 1D, Supplementary Table 1).

Two notable mutations remained after four backcrosses on Chromosome I: *hda-3(ix241)* and *dys-1(ix259). hda-3(ix241)* is a mutation in *hda-3* that leads to a glycine to glutamic acid substitution at the 271^st^ amino acid (G271E) (Figure 1E). *dys-1(ix259)* is a splice site mutation in *dys-1* (Figure 1F)*. hda-3* and *dys-*1 are located in close proximity on Chromosome I. OF1263, a strain which was backcrossed four times to the wild-type (WT) strain, and other backcrossed strains retained an exaggerated head bending phenotype. Exaggerated head bending has previously been observed in mutants with loss-of-function mutations to *dys-1*, the *C. elegans* ortholog of human Dystrophin (Figure 1G), and to genes encoding components of the Dystrophin associated protein complex (DAPC) [12–17]. We postulated that the exaggerated head-bending and the progressive decline in maximum velocity are linked and caused by the same underlying mutation. However, after the fifth backcross, we isolated OF1350, a strain that carried the *dys-1(ix259)* mutation and showed exaggerated head-bending, but did not show the progressive decline in maximum velocity and travel distance (Figure 1H, 1I; Supplementary Figure 1A, 1B). This result suggested that the *dys-1(ix259)* mutation is not sufficient to cause the locomotor deficit. This came as a surprise since Dystrophin, the human ortholog of DYS-1 is implicated in progressive muscle weakness [17, 18].

### *hda-3* mutation causes progressive decline in maximum velocity and travel distance

**Figure 2:**
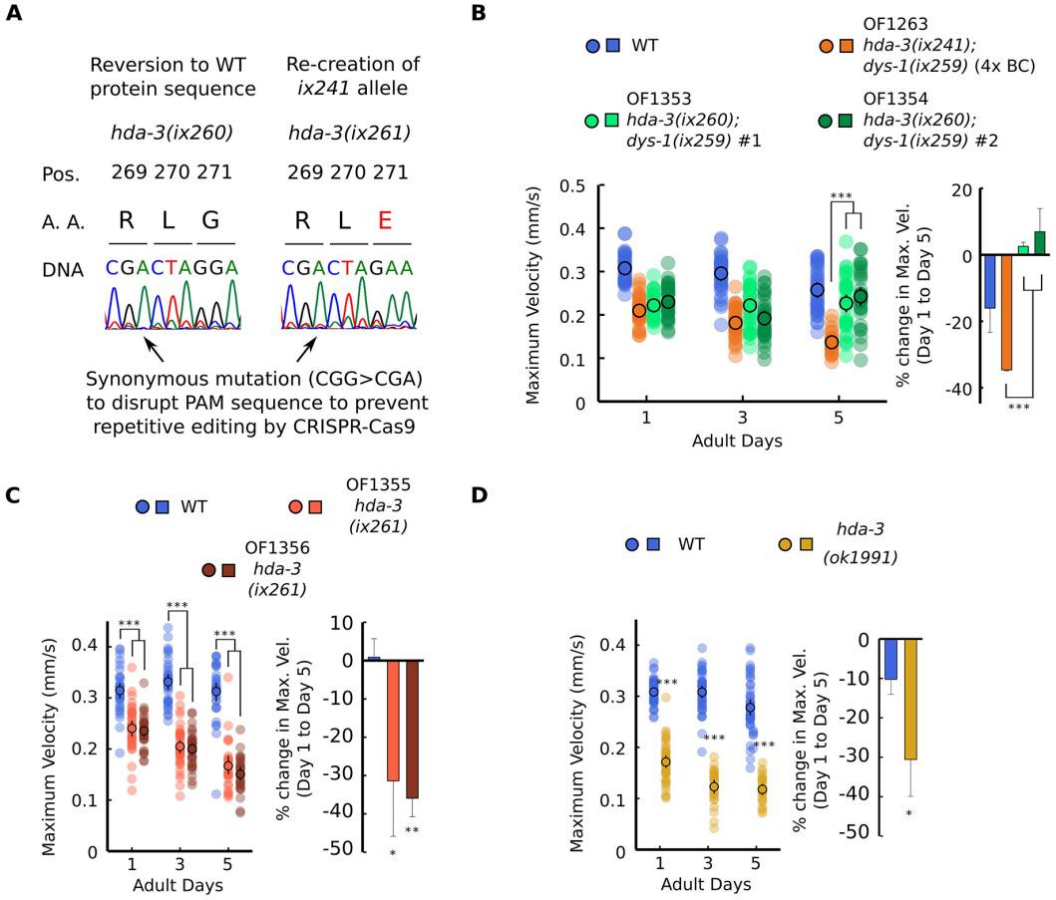
**HDA-3 G271E missense mutation leads to progressive decline in locomotor ability.** (**A**) (Left) Sequence of *hda-3(ix260)* allele which is the same amino acid sequence as WT. (Right) Sequence of *hda-3(ix261)* allele which is the same amino acid sequence as the *hda-3(ix241)* allele. Both sequences carry a synonymous mutation site to disrupt the PAM sequence to prevent repetitive editing by CRISPR-Cas9. (**B**) (*Left*) Maximum velocities of WT, OF1263 *hda-3(ix241);dys-1(ix259)* (4x BC), OF1353 *hda-3(ix260);dys-1(ix259)* and OF1354 *hda-3(ix260);dys-1(ix259)* worms. (*Right*) Percent change in maximum velocity of WT, OF1263, OF1353 and OF1354 worms on adult day 5 compared to adult day 1. (**C**) (*Left*) Maximum velocities of WT, OF1355 *hda-3(ix261)*, OF1356 *hda-3(ix261)* worms. (*Right*) Percent change in maximum velocity of WT, OF1355 *hda-3(ix261)*, OF1356 *hda-3(ix261)* worms on adult day 5 compared to adult day 1. (**D**) (*Left*) Maximum velocities of WT and *hda-3*(*ok1991)* worms. (*Right*) Percent change in maximum velocity of WT and *hda-3(ok1991)* worms on adult day 5 compared to adult day 1. For maximum velocity measurements, N = 30–45 worms per strain for each day (10–15 worms from 3 biological replicate plates). For percent change in maximum velocity graphs, N = 3 biological replicate plates. ***p < 0.001; **p < 0.01; *p < 0.05.

The effect of the *hda-3(ix241)* mutation was tested by two strategies. In the first strategy, CRISPR-Cas9 genome editing was used to revert the *hda-3(ix241)* mutation in the OF1263 *hda-3(ix241);dys-1(ix259)* strain (Figure 2A). In order to prevent repetitive editing, a synonymous mutation was introduced that would disrupt the protospacer adjacent motif (PAM) sequence, 5 bp upstream of the editing site (Figure 2A). We refer to this reverted allele as *hda-3(ix260),* which has the same HDA-3 amino acid sequence as WT HDA-3. In the second strategy, the HDA-3 G271E mutation was introduced into the WT N2 background using CRISPR-Cas9 genome editing. Again, a synonymous mutation was introduced that would disrupt the PAM sequence (Figure 2A). We refer to this mutation allele as *hda-3(ix261)*, which causes the same HDA-3 G271E mutation as the *hda-3(ix241)* mutation in the WT background. Strains carrying *hda-3(ix261)* were backcrossed twice.

The reversion of the *hda-3(ix241)* mutation to *hda-3(ix260)* rescued the progressive decline in maximum velocity and travel distance (Figure 2B, Supplementary Figure 2A). This result indicated that the *hda-3(ix241)* mutation is necessary for the progressive decline in maximum velocity and travel distance. In the *hda-3(ix261)* strain that carries the G271E mutation in the N2 WT background, progressive decline in maximum velocity and travel distance was observed (Figure 2C, Supplementary Figure 2B). This result indicated that the HDA-3 G271E mutation alone is sufficient to cause progressive decline in maximum velocity and travel distance. In addition, an independently isolated *hda-3(ok1991)* deletion strain showed progressive decline in maximum velocity and travel distance (Figure 2D, Supplementary Figure 2C). The reversion of the *hda-3(ix241)* mutation did not however reverse the minor locomotor deficit observed on the first day of adulthood (Figure 2B). Given that OF1350 *dys-1(ix259)* did not show a locomotor deficit on the first day of adulthood (Figure 1I), there may be other mutations in the background of OF1353 and OF1354 that lead to minor deficits on the first day of adulthood. Another possibility is that the synonymous codon mutation we introduced in the PAM site (CGG>CGA) for CRISPR genome editing may have unanticipated effects on the locomotor ability of worms.

**Figure 3:**
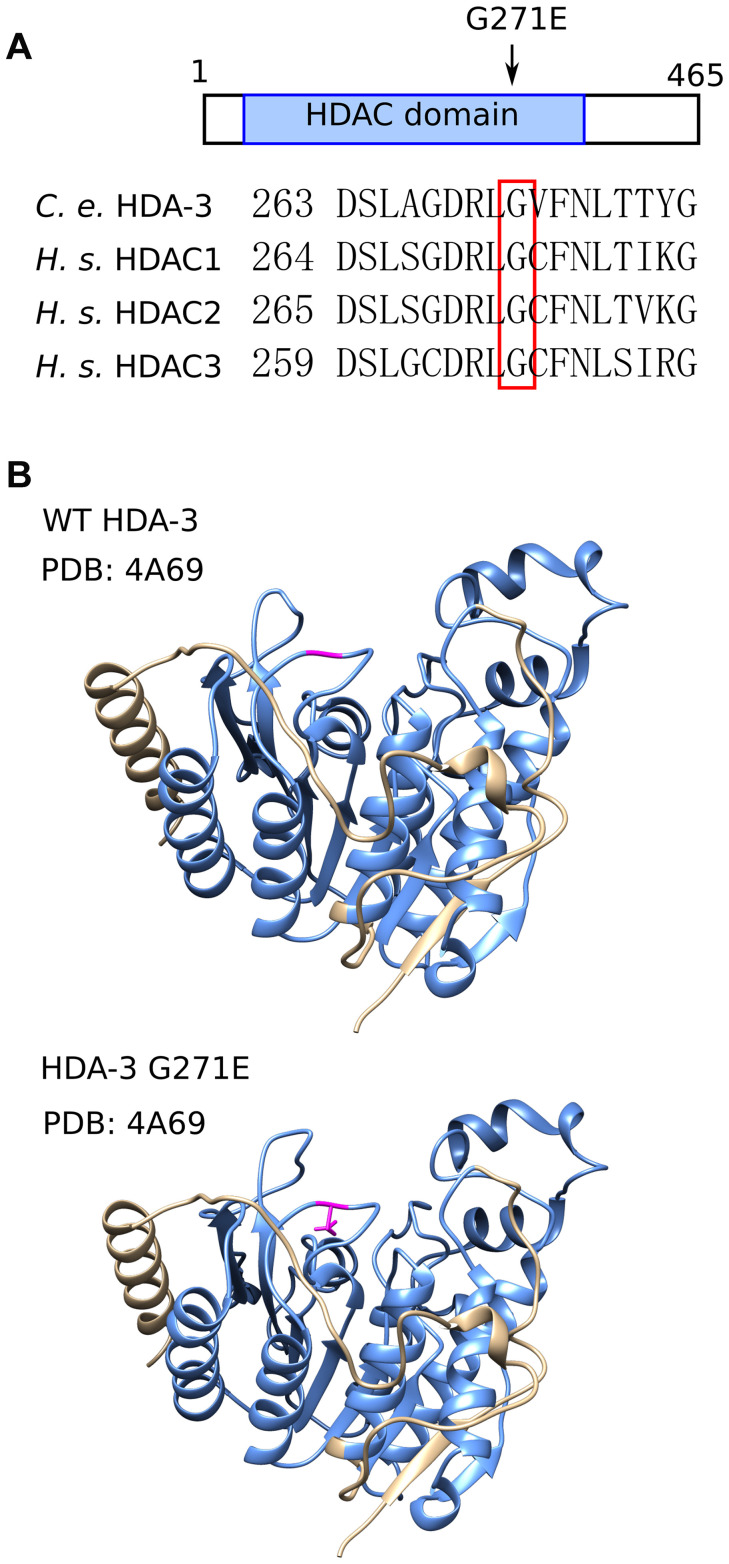
**HDA-3 G271E missense mutation occurs at evolutionarily conserved residue.** (**A**) (*Top*) Depiction of *hda-3(ix241)* mutation site in HDA-3 protein. (*Bottom*) Alignment of amino acid sequences centered around G271E mutation site in *C. elegans* HDA-3, *H. sapiens* HDAC1, HDAC2 and HDAC3. (**B**) (*Top*) Structural modeling of *C. elegans* WT HDA-3 based on PDB: 4A69 from *H. Sapiens* HDAC3. (*Bottom*) Structural modeling of *C. elegans* HDA-3 G271E based on PDB: 4A69 from *H. Sapiens* HDAC3. Mutated glutamic acid residue is shown in magenta. HDAC domain is indicated in blue.

The G271 residue is evolutionarily conserved from *C. elegans* to humans and is located in the variable loop region (Figure 3A, 3B; Supplementary Figure 3A). The variable loop region is suggested to play a role in substrate recognition and binding to the HDAC cofactors zinc and inositol phosphate [19, 20] (Figure 3B). The *C. elegans* G271 amino acid aligns with human HDAC3 G267. The HDAC3 G267 amino acid is in close proximity to R265, which is an amino acid that mediates the interaction between HDAC3 and its coactivator, inositol phosphate [19, 21]. HDAC3 D259 is also in close proximity which is predicted to mediate the interaction between HDAC family proteins with the cofactor zinc [20]. The G271E mutation may alter activities of HDA-3 that play a role in the maintenance of locomotor ability.

### Longevity is reduced in *hda-3* mutants

**Figure 4:**
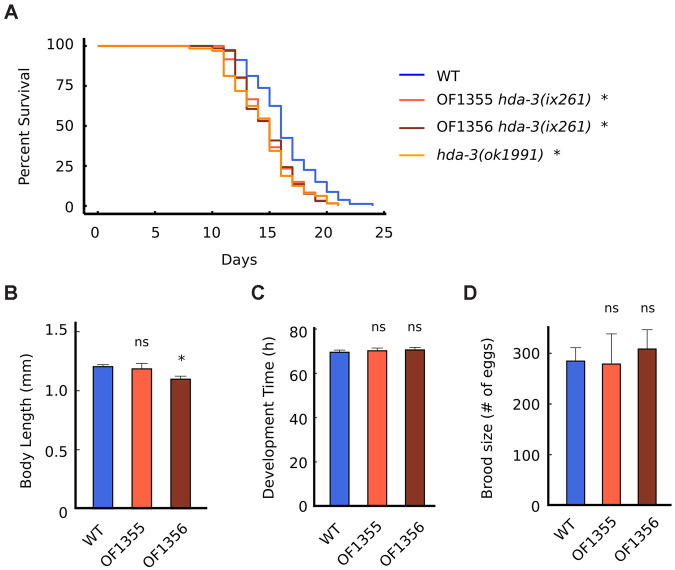
**HDA-3 mutation leads to shortened lifespan.** (**A**) Lifespan of WT (N = 80)*,* OF1355 *hda-3(ix261)* (N=60), OF1356 *hda-3(ix261)* (N=66), and *hda-3(ok1991)* worms (N = 64). (**B**) Body length of WT, OF1355 *hda-3(ix261)*, and OF1356 *hda-3(ix261)* worms. N = 10-17 worms per strain. (**C**) Development time of WT, OF1355 *hda-3(ix261)*, and OF1356 *hda-3(ix261)* worms. N = 8-10 worms per strain. (**D**) Brood size of WT, OF1355 *hda-3(ix261)*, and OF1356 *hda-3(ix261)* worms N = 5-6 worms per strain. *p < 0.05.

Since HDA-3 has previously been reported to be expressed ubiquitously [10], we tested whether mutation in HDA-3 may have effects on general aspects of health and longevity. The median lifespans of *hda-3(ix261)* mutants were reduced from 16 days to 15 days compared to WT worms (Figure 4A). The *hda-3(ix261)* mutants shared a comparable reduction in lifespan as *hda-3(ok1991)* mutant worms that carry a deletion in *hda-*3. The body length of the strain OF1355 *hda-3(ix261)* was unchanged compared to that of WT worms, but another strain, OF1356 *hda-3(ix261)* (Figure 4B), was shorter and may carry additional mutations besides *hda-3(ix261)* that have minor effects on body size. There were no significant differences in the development times and brood sizes of the *hda-3(ix261)* mutant worms and WT (Figure 4C, 4D).

OF1355 and OF1356 worms showed a 25 % reduction in survival 1 day earlier than WT worms (Figure 4A), while they showed a 25 % decline in maximum velocity 3 days earlier than WT worms (Figure 2C). This suggests that the negative effects of the hda-3(ix261) mutation affect maximum velocity more strongly than lifespan.

### Expression of subset of BATH genes are dysregulated in *hda-3* mutant strains

In order to identify gene expression changes that occur due to the *hda-3*(*ix241)* mutation, transcriptome analysis was carried out in adult worms on the third day of adulthood. On the third day of adulthood, WT worms did not show decline in maximum velocity and travel distance whereas *hda-3(ix241)* worms showed significant declines compared to the first day of adulthood (Figure 1I). Therefore, we reasoned that genes that play a role in the maintenance of adult maximum velocity and travel distance may be differentially expressed between WT and *hda-3(ix241)* worms on the third day of adulthood.

**Figure 5:**
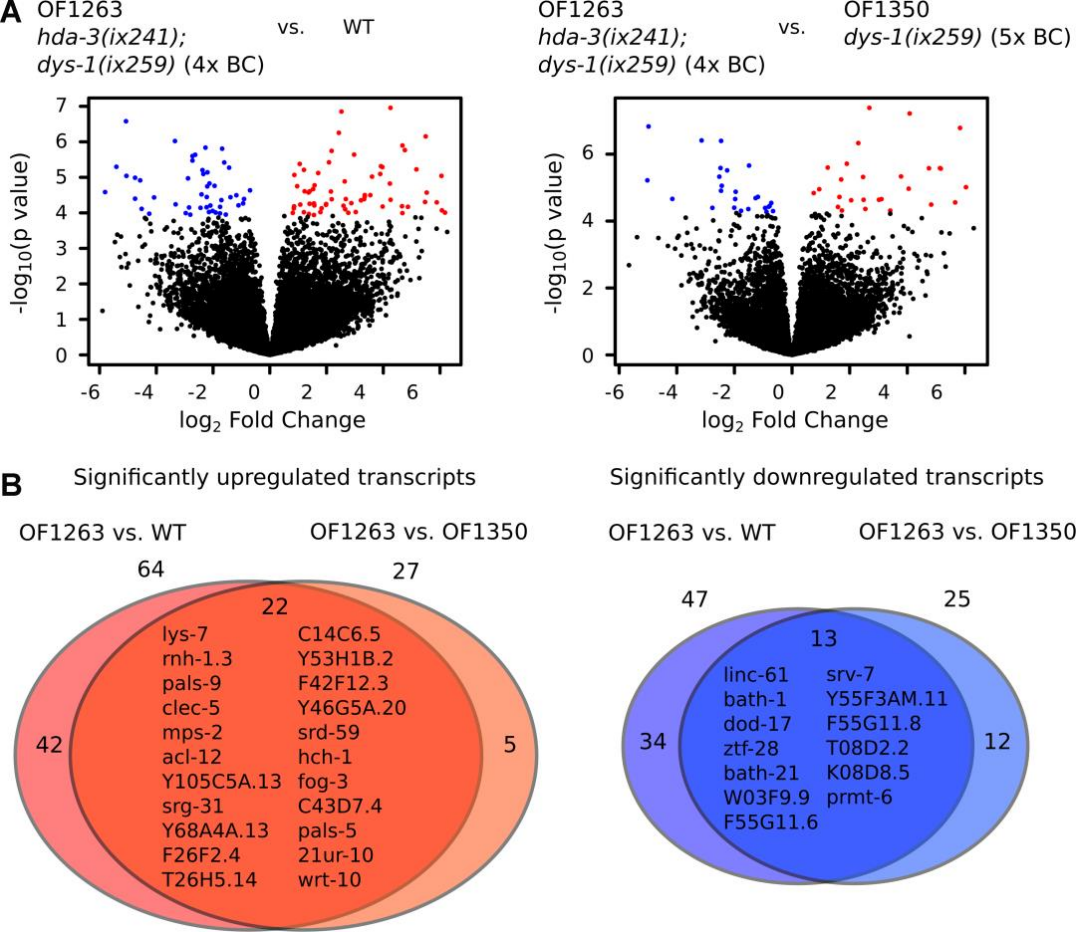
**Gene expression is dysregulated in *hda-3* mutant.** (**A**) (*Left*) Volcano plot of differential expression of transcripts from OF1263 *hda-3(ix241);dys-1(ix259)* (4x BC) vs. WT worms. (*Right*) Volcano plot of differential expression of transcripts from OF1263 *hda-3(ix241);dys-1(ix259)* (4x BC) vs. OF1350 *dys-1(ix259)* (5x BC) worms. Blue points indicate downregulated genes and red points indicate upregulated genes with *q* value < 0.05 (FDR-adjusted *p* value). (**B**) (*Left*) Venn diagram of number of significantly upregulated transcripts with *q* < 0.05 in OF1263 vs. WT worms and OF1263 vs. OF1350 worms. (*Right*) Venn diagram of number of significantly upregulated and downregulated transcripts with *P* < 0.0001 in OF1263 vs. WT and OF1263 vs. OF1350 worms. Names of commonly upregulated or downregulated genes are indicated within the Venn diagram.

In comparison to WT worms, OF1263 *hda-3*(*ix241);dys-1(ix259)* worms had 64 transcripts that were significantly upregulated and 47 transcripts that were significantly downregulated (FDR-adjusted *q* value < 0.05) (Figure 5A). In comparison to OF1350 *dys-1(ix259)* worms, which lost the *hda-3(ix241)* mutation after the fifth backcross, OF1263 worms had 27 transcripts that were significantly upregulated and 25 transcripts that were significantly downregulated (Figure 5A). Twenty-two transcripts were commonly upregulated in the OF1263 worms compared to WT and OF1350. Thirteen transcripts were commonly downregulated in the OF1263 worms compared to WT and OF1350 (Figure 5B).

**Figure 6:**
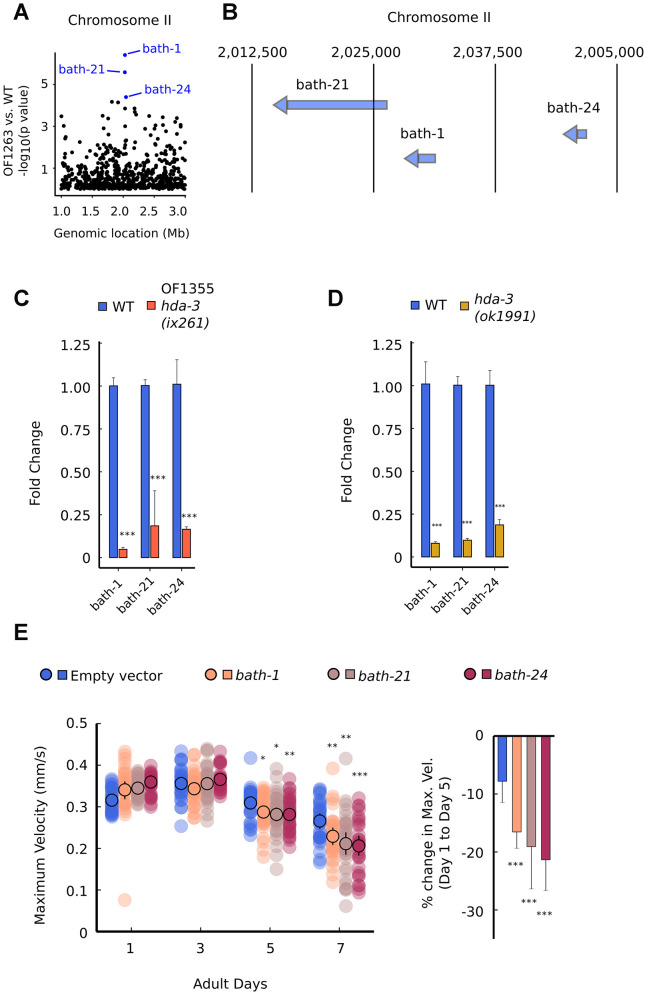
**Specific BATH genes on Chromosome II are downregulated from HDA-3 mutations.** (**A**) Genomic location of strongly downregulated gene transcripts on Chromosome II in OF1263 vs. WT. (**B**) Locations of BATH genes on Chromosome II. (**C**) Fold change in BATH genes in OF1355 *hda-3(ix261)* worms compared to WT, as measured by qPCR. (**D**) Fold change in BATH genes in *hda-3(ok1991)* worms compared to WT, as measured by qPCR. (**E**) Maximum velocities of worms fed with empty vector RNAi or RNAi targeted towards *bath-1, bath-21,* or *bath-24.* N = 30–45 worms per strain for each day (10–15 worms from 3 biological replicate plates). ***p < 0.001; **p < 0.01; *p < 0.05.

Among the downregulated transcripts, we noticed that multiple gene transcripts were downregulated within two narrow regions of the genome on Chromosome II and Chromosome IV (Figure 6A; Supplementary Figure 4A). On Chromosome II, the downregulated genes are *bath-1*, *bath-21*, and *bath-24* which carry BATH domains (Figure 6A, 6B) [22, 23]. BATH domains are characterized by BTB/POZ (broad-complex, Tramtrack and bric-a-bric/Pox virus and zinc finger) and MATH (meprin-associated Traf homology) domains [23]. Downregulation of *bath-1, bath-21* and *bath-24* were observed in both *hda-3(ix261)* mutant worms and *hda-3(ok1991)* mutant worms (Figure 6C, 6D). HDA-3 activity is likely to play a role in promoting expression of the chromosome region which encompasses *bath-1, bath-21,* and *bath-24.* In addition, a less stringent threshold for downregulation of genes (unadjusted *p* value < 0.05) indicates that *bath-3*, *bath-5*, and *btb-4* are also downregulated in the OF1263 strain (Supplementary Table 2). *btb-4* carries the BTB-domain, one of the two domains that characterize BATH genes. INTERPRO protein domain enrichment analysis of the differentially expressed genes (*p <* 0.05) indicates that BTB and MATH domains are most significantly enriched among downregulated genes (Supplementary Table 3). Interestingly, histone genes (*his-73, his-47, his-61, his-25, his-63, his*-*60*) that are constituents of the nucleosome are also a category of genes that are overrepresented among downregulated genes, but not in the upregulated genes (Supplementary Table 3). Precise regulation of the composition of histone variants may be carried out by HDA-3.

In order to test whether *bath* genes alone could have effects on motility, we knocked down *bath-1, bath-21,* and *bath-24* separately using feeding RNAi, and measured maximum velocity and travel distance. *bath-1, bath-21,* and *bath-24* individually had subtle but significant effects on maximum velocity and travel distance starting at the fifth day of adulthood (Figure 6E; Supplementary Figure 5A). These results suggest that *bath* genes may normally act to promote locomotor healthspan during later stages of reproductive adulthood.

## DISCUSSION

The role of class I HDACs during the aging process has been difficult to study, as HDAC1, HDAC2, HDAC3, and HDAC8 have been found to play important roles during development in vertebrates [24]. For example, HDAC3 knockout mice die during embryonic development [25]. HDACs have been linked to playing a role during the aging process as HDAC inhibitors have been shown to promote longevity in *C. elegans* and *D. melanogaster* [26–28]. HDAC inhibitors are also proposed to be therapeutic targets for various age-related pathologies such as sarcopenia and neurodegeneration [29–31].

Histone deacetylases can affect the transcriptional expression of many downstream genes [32]. Generally, histone acetylation is positively associated with transcriptional activation and deacetylation is associated with transcriptional repression [33]. However, other studies have found that HDACs are involved in both repression and activation [34, 35]. Our results show that *hda-3(ix241)* and *hda-3(ok1991)* mutations can lead to reduced expression of a subset of genes, and suggest that HDA-3 normally acts to promote expression of some genes. It is also possible that histone deacetylases may affect transcriptional outputs by mechanisms other than histone acetylation. HDAC3 can directly interact with transcription factors GATA-2 and Runx2 to repress their transcriptional activity [36, 37]. Histone deacetylases may affect transcriptional outputs by direct interactions or modifications to non-histone proteins.

Transcriptome analysis and quantitative PCR analyses of mutant strains carrying the HDA-3 G271E mutation indicated a region in the genome that is transcriptionally repressed on Chromosome II. The Chromosome II region carried genes of related function, *bath-1, bath-3, bath-5, bath-21, bath-24,* and *btb-4* that contain BTB/POZ and/or MATH domains [23]. Genes that contain both BTB and MATH domains are referred to as *bath* genes [23]. *bath* genes are reported to be involved in proteasomal degradation by association with CULLIN3 (CUL3), an E3 ubiquitin ligase [38, 39]. The BTB domain is reported to bind CUL3, while the MATH domain binds specific substrates to be degraded [38, 40]. Based on our study, *bath* genes may be part of the subset of genes that work to maintain locomotor healthspan during later stages of reproductive adulthood.

BATH-42 has been implicated in the regulation of nicotinic acetylcholine receptors by interacting with the CUL-3 ubiquitin ligase complex in *C. elegans* [41]. Expression of genes carrying BATH domains may be important for regulating the expression levels of other proteins that play a role in maintaining adult locomotor ability. As part of the ubiquitin-proteasome system, CUL-3 and BATH-domain carrying proteins may promote proteostasis by targeting misfolded proteins for degradation. *bath-24* was found to be enriched in *unc-4*::GFP expressing motor neurons, pointing towards a possible role in motor neuron functioning and locomotion [42]. *bath-1* and *bath-21* are reported to be enriched in germline, suggesting possible indirect roles in regulating healthspan [43]. Although the specific mechanism in which *hda-*3 and *bath* genes regulate locomotor healthspan is yet to be determined, they may do so by direct and indirect mechanisms originating from the neuromuscular system or other tissues.

The HDA-3(G271E) mutation occurs at an evolutionarily conserved residue. The same residue is present in human HDACs 1–3. The *C. elegans* G271 amino acid aligns with human HDAC3 G267. From exome and whole genome sequencing of the non-diseased human population, no missense mutations were reported in the HDAC3 G267 residue [44]. The G267 residue in human HDAC3 may be intolerant to amino acid changes, and thus play a critical role in proper HDAC3 function. The HDAC3 G267 amino acid is located in Variable Loop 4, one of the four variable loop regions which is implicated in substrate recognition [45]. The variable loops are different in length and sequence among the different HDAC proteins, enabling the recognition of specific substrates for each HDAC protein. Interestingly, all HDAC proteins, except for HDAC8, carry a glycine residue at the amino acid location aligned to HDAC3 G267. HDAC8 carries a cysteine residue instead of glycine. HDAC8 is believed to be evolutionarily divergent from the other class I histone deacetylases HDAC1-3 [46]. HDAC8 may have tissue-specific roles that are mediated by unique differences in amino acid sequence from the other HDAC proteins [47].

Since *C. elegans* can tolerate the loss of *hda-3* during development, the HDA-3 G271E mutants and *hda-3(ok1991)* deletion mutant may be valuable tools to study the role of a specific class I histone deacetylase during aging. Unbiased genomic screens have found that knockdown of *hda-3* leads to increased genome instability and plays a role in RNAi-mediated gene silencing [48]. The HDA-3 G271E mutants and *hda-3(ok1991)* deletion mutant have a reduced lifespan, and therefore may be used to identify genetic regulators of longevity downstream of HDA-3. Interestingly, Edwards et al. found that RNAi knockdown of *hda-3* leads to a 16% increase in lifespan for *C. elegans* [11]. Although the culture method in which *C. elegans* lifespan was measured differed between our study (agar plates) and Edwards et al. (liquid culture), partial reductions in HDA-3 function may have positive downstream gene expression changes for lifespan, while loss-of-function mutations to HDA-3 may have negative gene expression changes.

Genetic screens in *C. elegans* have identified various genes involved in locomotion. Upregulation of the EGF signaling pathway or downregulation of SLO-1 function have been shown to improve locomotion during adulthood [4, 5]. In a previous study, we identified a nonsense mutation in *elpc-2* that leads to progressive decline in maximum velocity and travel distance [6]. The role of *elpc-2* as part of the Elongator complex implicates the role of tRNA modifications for the maintenance of proteostasis and adult locomotor ability. In this study, we identify the G271E mutation in HDA-3 as the causative mutation site that leads to progressive decline in maximum velocity and travel distance. HDA-3 alters the transcription of BATH-domain genes located in close proximity on Chromosome II. In concert with the E3 ubiquitin ligase CUL-3, BATH-domain proteins may also regulate specific expression of proteins involved in motility, or affect proteostasis in a broader context. Together, these mutants provide insights into the genetic mechanisms that contribute to the maintenance of adult locomotor ability and may aid our understanding of the genetic bases of healthy aging.

## MATERIALS AND METHODS

### Strains

*C. elegans* Bristol N2 strain was used as the WT strain. Worms were cultivated at 20° C on Nematode Growth Media (NGM) agar plates with *Escherichia coli* strain OP50 as a food source [3]. All strains used in this study are listed in Supplementary Table 4.

### Edge assay and mutant isolation

The Edge Assay was performed as previously described [6]. In brief, Edge Assay plates were prepared by spreading 100 μL *E. coli* OP50 suspension near the edge of a circular 9 cm plate. “Synchronized egg-laying” was used to raise a batch of worms of similar age. Synchronized worms were collected with M9 buffer, and placed on the center of the Edge Assay plate. The Edge Assay completion rate was measured by counting the number of worms that reached the *E. coli* lawn near the edge of the plate divided by the total number of worms on the plate. The forward genetic screen to isolate worms with progressive decline in locomotor ability was performed using the Edge Assay as previously described [6]. In brief, L4 worms were mutagenized in 50 mM EMS solution for 4 h. Mutagenized F2 worms were placed on the center of Edge Assay plates on adult day 1. Worms that did not reach the edge within 15 minutes were removed by aspiration. Worms that reached the edge were maintained on the Edge Assay plate and tested again on adult day 3 and 5. The OF1262 strain was isolated as a worm that did not reach the edge on adult day 5. Isolated mutants that did not reach the edge were singled out on individual plates and maintained. Most worms produced at least a few progeny on adult day 5, but worms that did not produce progeny were abandoned.

### Sanger sequencing

Target genomic regions were amplified using PCR and purified using Wizard SV Gel and PCR Clean-Up System (Promega, Madison, WI). The DNA sequence of the PCR fragment was determined using cycle sequencing with BigDye v3.1 reagents (Applied Biosystems, Foster City, CA). Sequencing products were purified by EtOH/EDTA precipitation. 5 μL of 125 mM EDTA was added and mixed thoroughly to the 20 μL sequencing reaction. 60 μL of 100% ethanol was added and incubated for 15 min. The solution was centrifuged at 3000 x *g* for 30 min and the supernatant was discarded. 60 μL of 70% ethanol was added to the pellet and centrifuged at 1650 x *g* for 15 min. The supernatant was discarded, and the pellet was resuspended in 10 μL of Hi-Di Formamide (Applied Biosystems). Sequencing was performed by capillary sequencing using ABI3100 (Applied Biosystems). Primers used for Sanger sequencing are listed in Supplementary Table 5.

### Whole-genome DNA sequencing

*C. elegans* DNA was extracted by ethanol precipitation. Mixed-stage worms were collected using M9 buffer, and washed 3 times with lysis buffer (200 mM NaCl, 100 mM Tris-Hcl (pH 8.5), 50 mM EDTA, 0.5% SDS). 10 μL of 10 mg/mL proteinase K was added to the 500 μL lysis buffer solution containing the worms. The solution was incubated at 55° C for at least 3 h. Proteinase K was deactivated by incubation at 95° C for 30 min. 20 μL of 20 mg/mL RNAase A was added and incubated at 37° C for 30 min. 125 μL of saturated NaCl was added, and the solution was centrifuged for 15 min at 15,000 rpm. 500 μL of the supernatant was transferred to a new tube containing 1 mL of 100% ethanol. The solution was centrifuged at 15,000 rpm for 20 min. The supernatant was discarded and 1 mL of 70% ethanol was added to the pellet. The solution was centrifuged at 15,000 rpm for 20 min. The supernatant was discarded and the pellet containing DNA was reconstituted in 100 μL TE buffer. DNA was sequenced using the MiSeq next-generation sequencing system (Illumina, San Diego, CA) as previously described [6]. Libraries for sequencing were prepared with Illumina TruSeq Library Prep Kit. Sequenced reads were mapped using BWA software [49]. Mapped read files were converted to bam format, then to pileup format with Samtools [50]. Variant detection was carried out using VarScan and SnpEff [51–55]. Mutation effects were classified into four categories, (high, moderate, low, modifier) by SnpEff. The categorization of specific types of mutations are listed in the figure legend of Supplemetary Table 1. Mutations categorized as a high impact mutation were prioritized as most promising candidates that lead to the progressive decline in locomotion. Mutation frequencies were calculated and visualized using CloudMap [56].

### Measurements of maximum velocity and travel distance

Five adult day 1 worms were placed onto an NGM plate with food, and allowed to lay eggs for 3 h. When the offspring reached adult day 1, 15 worms were randomly picked onto a 6 cm NGM plate without bacteria. After the worms moved away from the initial location with residual food, worms were again moved onto a different NGM plate without bacteria. The maximum velocity and travel distance of worms were measured on the first, third, and fifth days of adulthood as previously described by video recording [6]. Videos were analyzed using ImageJ and wrMTrck software (www.phage.dk/plugins) to produce travel distance and maximum velocity values [57]. The same plate of worms measured on the first day of adulthood were measured on the third and fifth days of adulthood. For the percent change in maximum velocity or travel distance, the percentage decline from the first to fifth day of adulthood for each plate was calculated. Maximum velocity refers to the peak velocity that a worm reached during the 1.0 minute video recording. Travel distance refers to the total distance that the worm traveled during the 1.0-minute video recording. R was used to make plots [58].

### CRISPR-Cas9 genome editing

Targeted mutagenesis was carried out using CRISPR-Cas9 genome editing with single-stranded oligodeoxynucleotide (ssODN) donors as previously described [59]. First, a ribonucleoprotein complex was created by mixing together 0.5 μL of 10 μg/μL Cas9 protein, 5.0 μL of 0.4 μg/μL of tracrRNA, and 2.8 μL of 0.4 μg/μL of crRNA (Target-specific sequence: 5’-CCGAUUCACUGGCAGGAGAU-3’) and incubating at 37° C for 10 min. Following incubation, 2.2 μL of 1.0 μg/μL ssODN, 2.0 μL of 400 ng/μL pRF4::*rol-6(su1006)* co-injection marker, and 7.5 μL of nuclease free water was added to the mixture. This mixture was then injected into the gonad of young adult-stage worms subject to genomic editing. F1 offspring that showed the roller phenotype were singled onto individual plates, and allowed to lay eggs. Editing of the target sequence was checked by single worm PCR of the F1 worm, followed by Sanger sequencing. ssODN sequences are listed in Supplementary Table 6.

### Lifespan measurements

The lifespan of each worm strain was measured at 20° C. Worms that did not respond to prodding to the head and tail were considered dead. Worms that died from an exploded vulva, bag-of-worms phenotype, or that were lost during the incubation period were censored. Lifespan measurements were carried out in the absence of Floxuridine.

### Body length measurement

Photos of age-synchronized worms were taken on the first day of adulthood. Worms were mounted on agar pads in M9 buffer containing 20 mM sodium azide. A central line through the entire body of the worm was traced and measured using ImageJ [60].

### Brood size

Brood size was measured by allowing individual worms to lay eggs on a plate with food. The individual worm was moved to a fresh plate every 24 h until no more eggs were laid. The number of live larvae that were produced were counted on the next day. The total number of offspring that a single worm produced was counted as the total brood size.

### Development time

The time from egg to first egg-lay was considered as the development time of the worm. Gravid adults were allowed to lay eggs within a 1-h time window. Individual eggs were picked and placed on individual plates with food. Beginning at 66 h of development, worms were checked each hour to see if they had laid any eggs. The first time point in which eggs were laid was marked as the time of first egg-lay.

### RNA sequencing

Worms were synchronized by placing ten adult day 1 worms onto an NGM plate with food, and allowed to lay eggs for 3 h. At the L4 stage, worms were collected and washed with M9 buffer and placed on 9 cm NGM plates with 25 μM floxuridine (FUDR). On the third day of adulthood, worms were collected with M9 buffer and RNA was extracted. RNA was extracted by the phenol-chloroform method using Trizol reagent (Thermo Fisher Scientific). A 0.4 mL slurry of glass beads in water was added to 1 mL Trizol. Synchronized adult day 3 worms were collected with 500 μL M9 buffer, and the worms were allowed to settle to the tip of the pipette tip. A drop of M9 buffer containing the majority of worms at the tip of the pipette was dropped into the Trizol solution. Worms were homogenized by 4 sets of vortexing using Micro Smash MS-100R (Tomy Seiko, Tokyo, Japan) at 5,000 rpm for 2 min at 4° C. Additional vortexing cycles were carried out if worms were not completely homogenized. The solution was allowed to settle, and 800 μL of the supernatant was transferred to a new tube. 200 μL of Trizol and 200 μL of chloroform were added to the solution. The mixture was vortexed for 30 s and centrifuged at 15,000 rpm for 15 min at 4° C. 600 μL of the supernatant was added to a tube containing 600 μL 2-propanol. The solution was mixed by pipetting and incubated at room temperature for 10 min. This solution was centrifuged at 15,000 rpm for 15 min at 4° C. The supernatant was discarded, and 1 mL of 70% ethanol was added to the pellet. This solution was centrifuged at 10,000 rpm for 5 min at 4° C. The supernatant was discarded and the pellet was air-dried. RNA was reconstituted in 30 μL ddH2O. RNA sequencing was performed on the HiSeq platform (Illumina). For bioinformatics analysis, reads were aligned using STAR [61], sorting and marking duplicates were done by Picard, and read counting was done by Featurecounts [62]. EdgeR [63] and R [58] were used to create figures to visualize differential gene expression. For differential expression, an FDR-adjusted *q* value < 0.05 was used. In a less stringent analysis of differentially expressed genes, an unadjusted *p* value < 0.05 was used.

### Quantitative PCR (qPCR)

RNA was extracted from adult day-3 worms by the phenol-chloloroform method using Trizol reagent (Thermo Fisher Scientific) as described above. cDNA was synthesized using SuperScript III with oligo-dT primers (Thermo Fisher Scientific). 1 μL of oligo-dT primers, 1 μg of RNA, 1 μL of 10 mM dNTP mix and ddH20 was added to 13 μL. The solution was incubated at 65° C for 5 min and then on ice for 1 min. 4 μL of 5X First-Strand Buffer, 1 μL 0.1 M DTT, and 1 μL of SuperScript III was added to make 20 μL of solution. This solution was mixed by pipetting and incubated at 50° C for 60 min. The reaction was inactivated by heating at 70° C for 15 min. qPCR was carried out using the cDNA with Luna Universal qPCR Master Mix (New England Biolabs, Ipswich, MA) using StepOnePlus (Thermo Fisher Scientific). 100 ng of template cDNA was used for Luna Universal qPCR. The peroxisomal membrane protein *pmp-3* was used as the reference control for all qPCR experiments. Primers used for qPCR are listed in Supplementary Table 5.

### RNA interference

The Ahringer RNAi library was used to reduce the expression of target genes (*F59H6.8*, *F59H6.9*, *B0047.3*). Frozen stocks of the RNAi bacteria were streaked onto agar plates containing 100 μg/mL ampicillin. Single colonies of the RNAi bacteria were cultured for 8 h at 37° C with vigorous shaking in lysogeny broth (LB) containing 100 μg/mL ampicillin. 100 μL of bacterial culture was spread on NGM plates with 50 μg/mL ampicillin and 1.0 mM isopropyl β-D-1-thiogalactopyranoside (IPTG). Plates with RNAi bacteria were dried overnight with the lid on at room temperature (25° C). Adult wild-type worms were placed on RNAi plates and allowed to lay eggs for 3 h. The locomotor ability of the offspring was tested from the first day of adulthood. For mock control, an empty vector L4440 was used.

### Statistics

All results are expressed as means with error bars representing a 95% confidence interval. For pairwise comparisons, Student’s two-tailed *t* test was used with Excel 2010 (Microsoft). For multiple comparisons to a control, one-way ANOVA was followed with Dunnett’s post hoc test using R [58]. For multiple comparisons, one-way ANOVA was followed with Tukey’s Honest Significant Difference test using R [58]. For lifespan comparisons, log-rank test was used with R [58]. Statistical significance was set at *P < 0.05; **P < 0.01; ***P < 0.001.

### Data availability

All isolated strains are available upon request. DNA and RNA sequencing results are available on NCBI sequence read archive PRJNA530333.

## Supplementary Material

Supplementary Figures

Supplementary Tables
