# Supplementary material for: Mutation in histone deacetylase HDA-3 leads to shortened locomotor healthspan in Caenorhabditis elegans
Source: Aging (Albany NY). 2020 Dec 3;12(23):23525–47. doi: 10.18632/aging.202296 (PMC7762513; doi:10.18632/aging.202296)
Supplement: Supplementary Figures [file aging-12-202296-s001.pdf]

SUPPLEMENTARY FIGURES

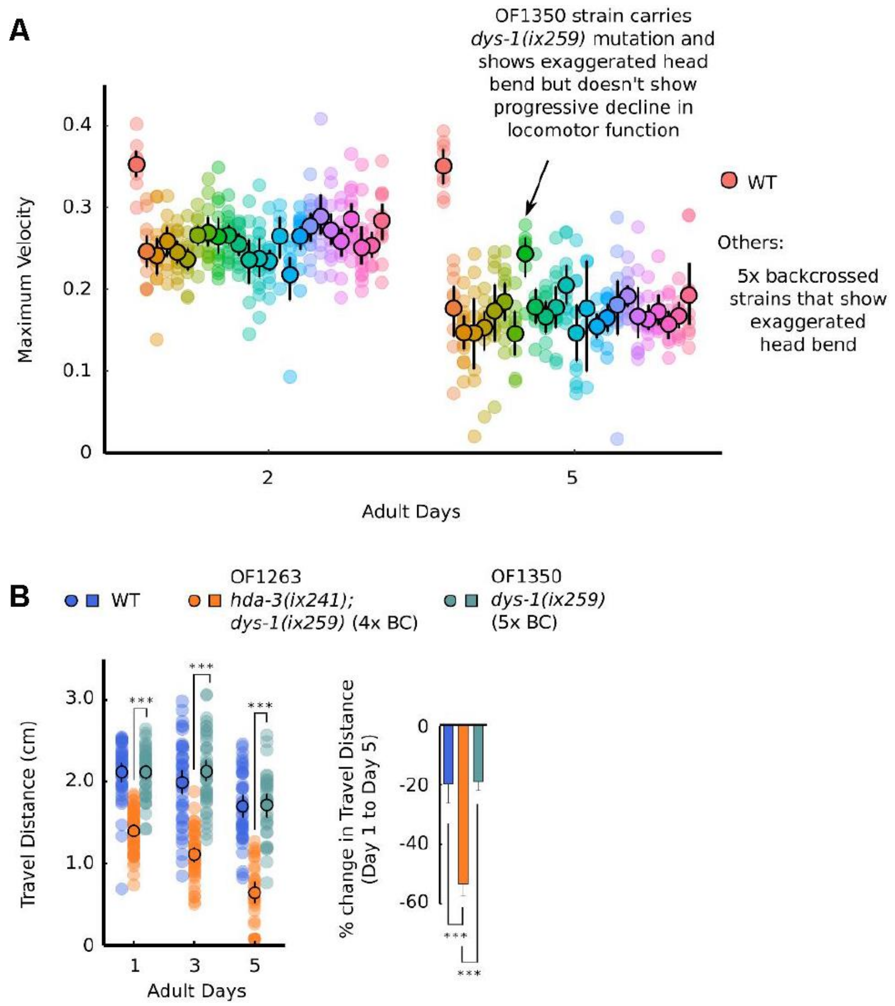

Supplementary Figure 1. *dys-1(ix259)* mutation does not cause progressive decline in locomotor ability. (**A**) Maximum velocities of 24 strains that show the exaggerated head bending phenotype after the fifth backcross on the second and fifth days of adulthood. N = 10–15 worms per strain. (**B**) (*Left*) Travel distances of WT, OF1263 *hda-3(ix241);dys-1(ix259)* (4x BC), and OF1350 *dys-1(ix259)* (5x BC) worms. N = 30–45 worms per strain for each day (10–15 worms from 3 biological replicate plates). (*Right*) Percent change in travel distance of WT, OF1263, and OF1350 worms. N = 3 biological replicate plates. ****P* < 0.001.

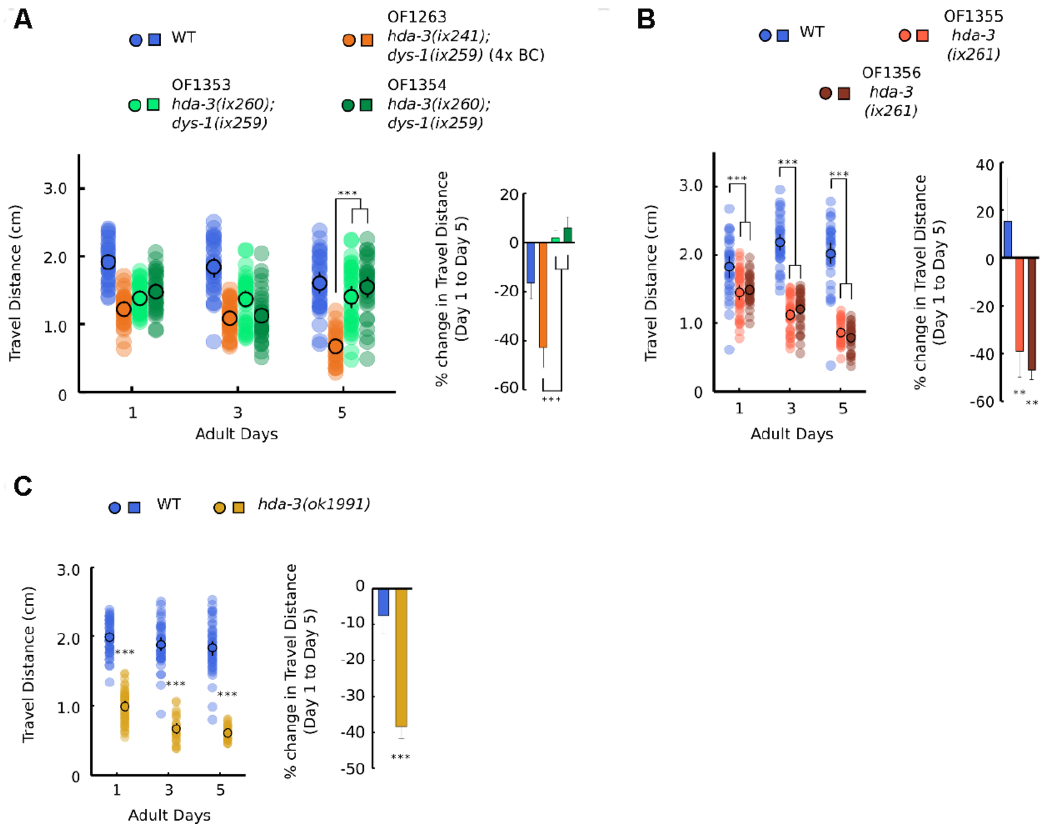

Supplementary Figure 2. *hda-3* mutation causes progressive decline in locomotor ability. (**A**) (*Left*) Travel distances of WT, OF1263 *hda-3(ix241);dys-1(ix259)* (4x BC), OF1353 *hda-3(ix260);dys-1(ix259)* and OF1354 *hda-3(ix260);dys-1(ix259)* worms on adult days 1, 3, and 5. (*Right*) Percent change in travel distance of WT, OF1263, OF1353, and OF1354 worms on adult day 5 compared to adult day 1. (**B**) (*Left*) Travel distances of WT, OF1355 *hda-3(ix261)*, and OF1356 *hda-3(ix261)* worms on adult days 1, 3, and 5. (*Right*) Percent change in travel distance of WT, OF1355 *hda-3(ix261)*, and OF1356 *hda-3(ix261)* worms on adult day 5 compared to adult day 1. (**C**) (*Left*) Travel distances of WT and *hda-3(ok1991)* worms on adult days 1, 3, and 5. (*Right*) Percent change in travel distance of WT and *hda-3(ok1991)* worms on adult day 5 compared to adult day 1. For travel distance measurements, N = 30–45 worms per strain for each day (10–15 worms from 3 biological replicate plates). For percent change in travel distance graphs, N = 3 biological replicate plates. ***$P < 0.001$; **$P < 0.01$.

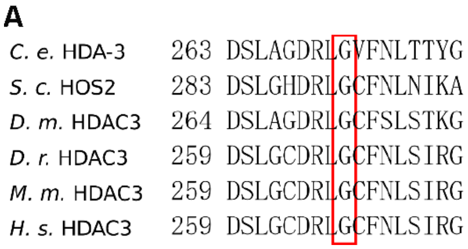

Supplementary Figure 3. Evolutionary conservation of HDA-3 G271 residue. (**A**) Alignment of *C. elegans* (C. e.) HDA-3 to *Saccharomyces cerevisiae* HOS2, *Drosophila melanogaster* HDAC3, *Danio rerio* HDAC3, *Mus Musculus* HDAC3, *and Homo sapiens HDAC3.*

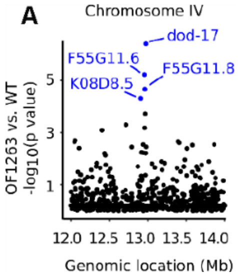

Supplementary Figure 4. Genes are downregulated in a narrow region on Chromosome IV. (**A**) Genomic location of strongly downregulated gene transcripts on Chromosome IV in OF1263 vs. WT.

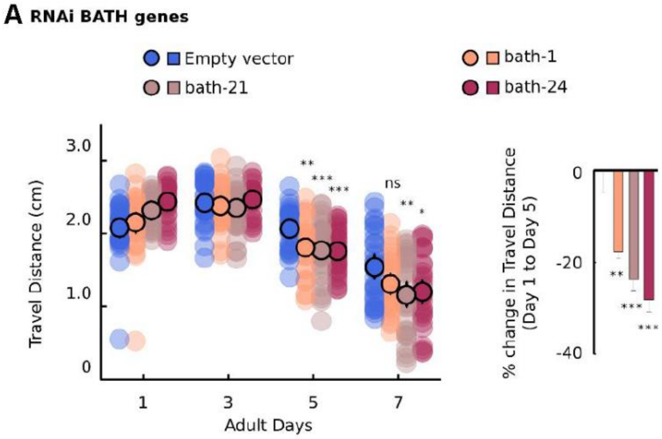

Supplementary Figure 5. Knockdown of BATH genes lead to progressive decline in travel distance. (**A**) Travel distances of worms fed with empty vector RNAi or RNAi targeted towards *bath-1, bath-21,* or *bath-24.* N = 30–45 worms per strain for each day (10–15 worms from 3 biological replicate plates). ***$p < 0.001$; **$p < 0.01$; *$p < 0.05$.
