# Supplementary material for: Mutation in histone deacetylase HDA-3 leads to shortened locomotor healthspan in Caenorhabditis elegans
Source: Aging (Albany NY). 2020 Dec 3;12(23):23525–47. doi: 10.18632/aging.202296 (PMC7762513; doi:10.18632/aging.202296)
Supplement: Supplementary Tables [file aging-12-202296-s002.pdf]

SUPPLEMENTARY TABLES

Supplementary Table 1. List of remaining mutations in backcrossed strains.

Chrom.	Pos.	Ref.	Alt.	Gene	Mutation type	Effect*
I	3258592	CA	C	*Y54E10A.20*	upstream gene variant	modifier
I	8160530	TTATA	T	*T28B8.3*	downstream gene variant	modifier
I	10731034	C	T	*rpn-10*	missense variant	moderate
I	10766899	C	T	*daf-16*	intron variant	modifier
I	10974485	C	T	*Y52B11A.3*	intron variant	modifier
I	11307424	C	T	*H25P06.5*	synonymous variant	low
I	11536456	C	T	*dys-1*	splice acceptor variant	high
I	11644705	C	T	*W04G5.9*	intron variant	modifier
I	11726250	C	T	*F35E2.9*	missense variant	moderate
I	11808061	G	T	*T02G6.2-T02G6.4*	intergenic region	modifier
I	11832340	C	T	*Y47H9C.1*	missense variant	moderate
I	11864150	T	A	*ced-1*	intron variant	modifier
I	11896398	C	T	*Y47H9C.12*	upstream gene variant	modifier
I	11914682	C	T	*hda-3*	missense variant	moderate
I	11927975	C	T	*wve-1*	upstream gene variant	modifier
I	12008815	C	T	*fbxa-122*	downstream gene variant	modifier
I	12176794	C	T	*R05D7.3*	intron variant	modifier
I	12298210	C	T	*F56H6.7*	missense variant	moderate
I	12341691	C	T	*nhr-217*	intron variant	modifier
I	12343381	T	A	*T09E11.11*	upstream gene variant	modifier
I	12414199	C	T	*E03H4.5*	intron variant	modifier
I	12493515	C	T	*T27F6.6*	synonymous variant	low
I	12970406	G	A	*eif-6*	downstream gene variant	modifier
I	14083393	A	C	*gadr-6*	upstream gene variant	modifier
III	2340737	A	C	*Y54F10BM.1*	intron variant	modifier
III	3385124	A	AG	*hecw-1*	upstream gene variant	modifier
III	3786851	T	TTC	*acy-3*	upstream gene variant	modifier
III	6301707	T	TC	*F47D12.9*	upstream gene variant	modifier
IV	1226960	T	C	*W09G12.8*	intron variant	modifier
IV	12319292	TG	T	*F19B6.9*	downstream gene variant	modifier
IV	12319295	T	A	*F19B6.9*	upstream gene variant	modifier
IV	13506453	GA	G	*nlp-17*	upstream gene variant	modifier
IV	13823462	A	AACTCGGCTGTCG GCTGGCGCCGACA GCCGAGTCCATTTC GCT	*H08M01.74*	downstream gene variant	modifier
V	363225	C	CTACTGTAGTGCTT GTGTCGATTTACGG GATCGATTTCCTAA ATGAACCGTAAATC GACACAAGCACTA CAGTAGTCATTTAA AGGAT	*T22H9.1*	intron variant	modifier
V	1500601	G	A	*sru-27*	missense variant	moderate
V	13231585	T	G	*C34D1.8*	downstream gene variant	modifier
V	13697695	C	T	*T01D3.1*	missense variant	moderate
X	1519515	A	ATCCGACATTTTTA TAGCAATGCGCAG AACCCAAAAAATG TCGGACGCGGAGC CAAGGCTGCACCA AATAGTGCGATAG GGTATGGCATTATT TGGTGCAAACTTG GCTTCGCG	*toca-1*	intron variant	modifier
X	2008595	TA	T	*Y40A1A.1-Y102A11A.9*	intergenic region	modifier
X	3314891	A	AT	*F11D5.12*	upstream gene variant	modifier
X	4241974	A	G	*W01H2.t1*	upstream gene variant	modifier
X	4674025	G	GT	*F16H11.1*	downstream gene variant	modifier
X	6357781	C	CCCAT	*C03B1.10*	frameshift variant	high
X	8191585	G	GT	*C17H11.6*	intron variant	modifier
X	11646516	A	C	*T04F8.15*	upstream gene variant	modifier

X	12502801	T	TACGAAAAATAGA TTGTTAC	*sdz-19*	intron variant	modifier

Candidate causative mutation sites for the OF1262 strain.
*Putative impact of mutation as categorized by snpEff: high (splice site variant, frameshift variant) > moderate (missense variant) > low (synonymous variant) > modifier (upstream gene variant (5'UTR), downstream gene variant (3'UTR), intron variant, intergenic variant).

Supplementary Table 2. Average read counts and *p* values of downregulated region on Chromosome II.

Gene	Chr	Start	Strand	Avg Read Count WT	Avg Read Count OF1263	Avg Read Count OF1350	*p* value OF1263 vs. WT	*p* value OF1263 vs. OF1350
F59H6.3	II	2017450	+	544.3333333	503.75	617.5	0.850896676	0.560942062
F59H6.2	II	2019654	+	46.33333333	37.25	57	0.945386699	0.48335894
cya-2	II	2022673	-	647.6666667	145.75	342.25	0.000111143	0.017196088
bath-21	II	2014902	-	500.3333333	85.75	492.5	0.00000122	0.00000011
bath-1	II	2027612	-	1753	233	2190.75	2.69E-10	5.06E-14
bath-3	II	2029236	-	548.6666667	248.5	902	0.044371025	0.000061
bath-5	II	2030753	-	7225.666667	2460.75	8269.75	0.000945556	0.0000203
btb-4	II	2032316	+	2886	456.5	1612	0.00000698	0.000828007
F59H6.14	II	2033586	+	48.33333333	15	23.25	0.025843936	0.342383526
F59H6.13	II	2034351	-	0	0	0	1	1
bath-19	II	2035695	-	1246	730.25	1966.25	0.30014777	0.004494907
bath-20	II	2040276	-	101	40.75	77.5	0.104265019	0.179052293
btb-3	II	2043089	-	157.3333333	95.5	249.75	0.456879336	0.022200842
bath-24	II	2044619	-	1507.333333	412.5	1697.5	0.0000992	0.0000024
math-1	II	2046053	-	27.66666667	8.5	17	0.182230378	0.324120223
bath-14	II	2048061	-	218	84.5	301.75	0.065982827	0.001813642
F07E5.4	II	2050023	-	0	0	2	1	0.199767663
fbxb-35	II	2051672	+	43.66666667	151.75	109.5	0.00256399	0.393136077
F07E5.5	II	2053919	-	6974.666667	7446.25	9206.5	0.400658623	0.389501995

Supplementary Table 3. INTERPRO analysis of protein domain enrichment in genes downregulated in OF1263 strain vs. OF1350 and WT.

Category	Term	Count	%	PValue	Genes
INTERPRO	IPR002083:MATH	8	3.61991	3.62E-05	WBGENE00020532, WBGENE00019139, WBGENE00015014, WBGENE00019138, WBGENE00018223, WBGENE00022690, WBGENE00019141, WBGENE00019140
INTERPRO	IPR000210:BTB/POZ-like	7	3.167421	0.004653	WBGENE00019139, WBGENE00015014, WBGENE00019138, WBGENE00018223, WBGENE00019142, WBGENE00019141, WBGENE00019140
INTERPRO	IPR002486:Nematode cuticle collagen, N-terminal	9	4.072398	4.20E-04	WBGENE00000712, WBGENE00000615, WBGENE00000703, WBGENE00001066, WBGENE00000660, WBGENE00000256, WBGENE00000685, WBGENE00000753, WBGENE00000678

INTERPRO	IPR001304:C-type lectin	9	4.072398	0.003387	WBGENE00021586, WBGENE00021873, WBGENE00014063, WBGENE00021587, WBGENE00009397, WBGENE00012583, WBGENE00007805, WBGENE00020191, WBGENE00009517
INTERPRO	IPR013781:Glycoside hydrolase, catalytic domain	4	1.809955	0.039608	WBGENE00016340, WBGENE00020509, WBGENE00016335, WBGENE00044807
INTERPRO	IPR008758:Peptidase S28	3	1.357466	0.006103	WBGENE00017594, WBGENE00003959, WBGENE00019682
INTERPRO	IPR009072:Histone-fold	6	2.714932	0.003181	WBGENE00001935, WBGENE00001947, WBGENE00001934, WBGENE00001937, WBGENE00001921, WBGENE00001899

Supplementary Table 4. List of strains used in this study.

Strain	Genotype	Obtained from
OF1262	*hda-3(ix241);dys-1(ix259) I*	Isolated in previous study (Kawamura and Maruyama, 2019); Also referred to as *ix241*
OF1263	*hda-3(ix241);dys-1(ix259) I* (4x backcrossed)	Isolated in previous study (Kawamura and Maruyama, 2019); Also referred to as *ix241*(4x BC)
OF1350	*dys-1(ix259) I* (5x backcrossed)	This study
OF1353	*hda-3(ix260);dys-1(ix259) I* #1	This study. Independent CRISPR strain from OF1354 (Same injection mixture).
OF1354	*hda-3(ix260);dys-1(ix259) I*; #2	This study. Independent CRISPR strain from OF1353 (Same injection mixture).
OF1355	*hda-3(ix261) I* (2x backcrossed) #1	This study. Independent CRISPR strain from OF1356 (Same injection mixture).
OF1356	*hda-3(ix261) I* (2x backcrossed) #2	This study. Independent CRISPR strain from OF1355 (Same injection mixture).
RB1618	*hda-3(ok1991) I*	CGC
CF1038	*daf-16(mu86) I*	CGC
PS3551	*hsf-1(sy441) I*	CGC
LS292	*dys-1(cx18) I*	CGC
BZ33	*dys-1(eg33) I*	CGC

List of strain, genotype, and source of *C. elegans* used in this study.

Supplementary Table 5. List of primers used in this study.

Primer name	5'-3' Sequence
dys-1(ix259) 5'	atgggcatgatgggtgtcaaatgaa
dys-1(ix259) 3'	cagaaaggcttccaccagtcggttg
hda-3(ix241) 5'	ggaatttgaaatttccggcaaatgtgcgaatggca
hda-3(ix241) 3'	tccacgaggagtacacgagagcttcttcgtaa
bath-1 qpcr 5'	ggttatcgatgatgatgacgtg
bath-1 qpcr 3'	gagacaagactttttcaaattgtcc
bath-21 qpcr 5'	ttctcagaaagttccttgcctc
bath-21 qpcr 3'	caaccgtgtcatcatctatagc
bath-24 qpcr 5'	tgcgattgatgattctaccatcg
bath-24 qpcr 3'	gagaggcaaacggttttcaaatt

pmp-3 qpcr 5’	tggccggatgatggtgtcgc
pmp-3 qpcr 3’	acgaacaatgccaaaggccagc

List of primers used for genotyping and qPCR.

Supplementary Table 6. List of single-stranded oligodeoxynucleotide sequences used in this study.

ssODN name	5'-3' sequence
hda3(ix241) repair to WT	CCATAAGTAGTCAAATTGAATACTCCTAGTCGATCTCCTGCCAGTGAATCGGCACCACATTGGAGCACAA
hda3(ix241) mutation introduction	CCATAAGTAGTCAAATTGAATACTTCTAGTCGATCTCCTGCCAGTGAATCGGCACCACATTGGAGCACAA

List of single-stranded oligodeoxynucleotide sequences used to repair the *hda-3(ix241)* allele to WT and to re-introduce the *hda-3(ix241)* allele into the WT background.
